# Quantifying Reliable Walking Activity with a Wearable Device in Aged Residential Care: How Many Days Are Enough?

**DOI:** 10.3390/s20216314

**Published:** 2020-11-05

**Authors:** Christopher Buckley, Alana Cavadino, Silvia Del Din, Sue Lord, Lynne Taylor, Lynn Rochester, Ngaire Kerse

**Affiliations:** 1 Department of Sport, Exercise and Rehabilitation, Faculty of Health and Life Science, Northumbria University, Newcastle Upon Tyne NE1 8ST, UK; christopher.buckley@northumbria.ac.uk; 2 Translational and Clinical Research Institute, Faculty of Medical Science, Newcastle University, Newcastle Upon Tyne NE1 7RU, UK; silvia.del-din@ncl.ac.uk (S.D.D.); lynn.rochester@ncl.ac.uk (L.R.); 3 School of Population Health, Faculty of Medical and Health Sciences, The University of Auckland, Auckland 1023, New Zealand; a.cavadino@auckland.ac.nz (A.C.); lm.taylor@auckland.ac.nz (L.T.); 4 School of Clinical Sciences, Auckland University of Technology, Auckland 1010, New Zealand; sue.lord@aut.ac.nz; 5 The Newcastle upon Tyne Hospitals NHS Foundation Trust, Newcastle Upon Tyne NE7 7DN, UK

**Keywords:** aged residential care, wearables, accelerometer, dementia, reliability, physical activity

## Abstract

Strong associations exist between quality of life and physical activity for those living in aged residential care (ARC). Suitable and reliable tools are required to quantify physical activity for descriptive and evaluative purposes. We calculated the number of days required for reliable walking outcomes indicative of physical activity in an ARC population using a trunk-worn device. ARC participants (*n* = 257) wore the device for up to 7 days. Reasons for data loss were also recorded. The volume, pattern, and variability of walking was calculated. For 197 participants who wore the device for at least 3 days, linear mixed models determined the impact of week structure and number of days required to achieve reliable outcomes, collectively and then stratified by care level. The average days recorded by the wearable device was 5.2 days. Day of the week did not impact walking activity. Depending on the outcome and level of care, 2–5 days was sufficient for reliable estimates. This study provides informative evidence for future studies aiming to use a wearable device located on the trunk to quantify physical activity walking out in the ARC population.

## 1. Introduction

In order to understand mobility in aged residential care (ARC) and the efficacy of interventions that may change mobility, it is imperative that measurement of outcomes indicative of physical activity are feasible and reliable [1,2]. Previously, research concerning patterns of physical activity for ARC residents relied on interviews or self-report questionnaires [3]. The objectivity of these methods is reduced because of reliance on recall. As an alternative, studies have begun to monitor physical activity using wearable devices in low level care (i.e., assisted living and care homes) [2,4,5,6,7,8,9] and higher level care (i.e., nursing homes) settings [10,11]. However, protocols for data collection vary, with the number of days for data collection ranging from 2 to 14 days, often with no justification for their duration [12]. Recording data for fewer days has obvious practical advantages. In the ARC setting, compliance for prolonged device use could be lower for residents with varying levels of cognition and care needs. Participant burden is also less and, in the ARC setting, so are the demands on research or facility staff to regularly check the devices. Fewer days also reduces the time required to process data. Determining the number of days to reliably estimate physical activity walking outcomes in ARC is therefore important to optimize compliance and data quality

Day-to-day variability in physical activity is lower in older adults than other age groups, meaning that less days of recording may be required, depending on the outcome of interest [13]. Rowe et al. [13] reported inter-day reliability of 0.87 (95% confidence intervals, 0.79–0.92) for step counts with only 2 days of data in 62 community dwellings of older adults using a waist-worn accelerometer. Egerton et al. [10] found a within-subject reliability intra-class correlation coefficient (ICC) of 0.92 (SEM 0.26) for upright time with 3 days’ data, using a thigh-worn device in a pilot study in community dwellers. They also noted that weekday activity did not differ from weekend activity. Van Schooten et al. [1] assessed the number of days required to measure body postures and walking using a trunk-worn accelerometer in 102 older adults that included 23 participants from ARC (additional analysis confirmed little difference between ARC residents and community dwellers). Results indicated that, to achieve a between day ICC > 0.7 at a group level, a minimum of 2 days was required for most activities, while outcomes, such as lying and median duration of locomotion bouts, required up to 5 days.

However, none of these studies inform about older adults in ARC who present with advanced cognitive impairment and have much lower levels of mobility. Analysis of the use of wearable devices in ARC needs to take into account how many days are required for reliable data, as well as how well the wearable device is tolerated.

The aims of this study were, firstly, to examine the number of days required for reliable estimates of physical activity walking outcomes for residents living in ARC using a single wearable device while controlling impact of week structure and level of care and, secondly, to describe the compliance to wearing a trunk-worn device continuously for 7 days.

## 2. Materials and Methods

### 2.1. Design

This study used baseline data from the “Staying UpRight in residential care”, a randomized controlled trial that evaluates the effects of an exercise program on fall rates of older adults in ARC [14]. The primary outcome was fall rates and a secondary outcome was fall rate relative to activity exposure, expressed using step counts and measured using a wearable device, as described below. Participants were recruited from 17 ARC facilities in Auckland and Hamilton, Aotearoa, and New Zealand (NZ). The study was approved by the national health and disability ethics review board. Consent was obtained from participants or registered nursing staff for those unable to consent, in accordance with NZ ethics requirements.

### 2.2. Participants

ARC residents aged 65 years or older, classified as requiring (i) intermediate-level care, i.e., 24 h health-related care and services but not 24 h nursing care; (ii) high-level care residents, i.e., 24 h nursing care; or (iii) dementia-level care, i.e., 24 h health-related care in a secure environment due to risk of wandering because of memory loss, were invited to participate. Residents in psychogeriatric, respite, or palliative care and acutely unwell or immobile (bed-bound) residents were excluded.

### 2.3. Instrumentation

Participants wore a wearable device (wearable) containing a tri-axial accelerometer (Axivity AX3, Axivity, York, UK), secured on the lower back at the fifth lumbar vertebrae (L5) using a hydrogel adhesive (PALStickies, PAL Technologies, Glasgow, UK or Smith + Nephew Ltd., Watford, UK), covered with an adhesive dressing (OPSITE Flexifix™ (Amgel Technologies, Portland, OR, USA) and Hypafix™ (BNS medical, Hamburg, Germany). The wearable was programmed to sample acceleration at a frequency of 100 Hz (range ± 8 g) and was applied by trained research staff to ensure the correct application.

### 2.4. Wearable Data Analysis

Upon the retrieval of the wearable, the participant’s data over a total of 8 days (half days for the first and final day, creating a total of 7 days of wear time) were downloaded from the wearable before then being uploaded to an encrypted, secure platform (eScience Central online platform, Newcastle University, UK) [15,16,17]. The online platform was used to remotely segment the data into days in separate .mat files. Following download, data were visually inspected by a trained technician. Data that did not meet the quality check criteria, such as movements not possible by humans, movements of the research staff upon retrieving devices asked to be removed/fallen off, evident misplacement of the sensor (not aligned to the earth’s gravitational constant), or completely stationary signals (such as a removed monitor that had been placed aside), were removed to avoid including erroneous data into subsequent analysis.

**Figure 1:**
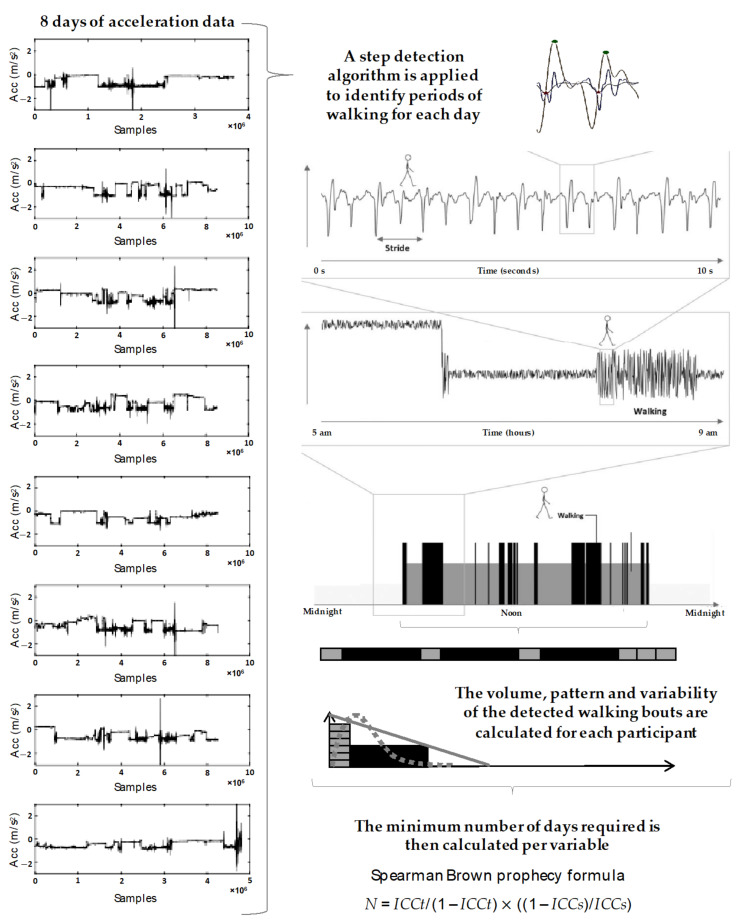
A flow diagram indicating the data processing pipeline, where 8 days of raw acceleration signal is refined to be included in the calculation of the minimum days required for each variable. Adapted with permission from [19].

To answer the first aim, a custom automated MATLAB algorithm calculated physical activity walking outcomes according to a previously established gait model, described in detail elsewhere [18,19,20,21,22,23]. The model quantifies the volume, pattern, and variability of walking behaviors. Volume metrics included total walking time, total steps, and total bouts per day. The pattern included mean walking bout length, generated based on the ambulatory bouts (ABs), and a non-linear descriptor (alpha). Alpha describes ABs distribution, evaluating the ratio of short to long ABs (i.e., a high alpha means that the total walking time is made up of proportionally short ABs compared to long ABs) [24]. Variability of walking bouts was derived from the “within-subject” variability of AB length, with a higher variability, indicating a more varied walking activity pattern, and a lower variability (S2) would mean a less varied walking activity, thus a reduced engagement in different activity and a tendency to repeat the same pattern of activity [20,25]. Figure 1 indicates the process of calculating the volume, pattern, and variability for a single participant from data collected over an 8-day period.

To address the second aim, reasons for any data loss were recorded for each participant.

### 2.5. Data Considerations

All outcomes were calculated for each day and for walking bouts greater than 3 steps, which was considered the minimum bout length for analysis [20,26,27,28]. A threshold of 2.5 s was set for the maximum resting period between consecutive ABs [23]. Due to data recording being initiated on varied weekdays, the data was reordered into a uniform week structure starting on Monday. Averages were calculated for weekdays and weekends (Saturday and Sunday) to control for any weekday/weekend effects on activity, in addition to the effect of facilities where the data were collected. Only participants who had a least 3 full days of analysis (removing days one and eight for being half days) were included to calculate the number of days required for reliable estimates of each outcome, in order to achieve an adequate representative within person variance. To determine the impact of level of care, all analysis was performed and stratified by an intermediate, high, and dementia unit level of care.

### 2.6. Statistical Analysis

All analyses were performed using Stata, version 16 (Stata, TX, USA). Data are presented using medians and interquartile ranges (IQRs) due to non-normal outcome distributions. Mean walking bout length, total walking time, total steps, and total bouts per day were analyzed using a square root transformation to improve the normality of residuals. Variability and alpha of walking bouts was analyzed on their original scale.

Linear mixed models with a random intercept for individuals and post hoc pairwise multiple comparisons were used to determine differences between any pair of days in the week. A *p*-value threshold of 0.01 was used to account for multiple comparisons. Potential week/weekend differences were also evaluated by including a dummy variable for week vs. weekend day.

The ICC is a reliability index that reflects the consistency and degree of correlation between measurements, with values above 0.8 generally considered to indicate a good or acceptable level of reliability. The minimum number of days needed to obtain an ICC of at least 0.8 was calculated for each outcome, based on the methods highlighted in [29]. In short, the reliability for single days of measurement was assessed using variance partitioning obtained through linear mixed effects models with random intercepts for participant ID, reporting the ratio of the between subject variance to the total variance (between subject variance/(between subject variance + residual variance)). For analysis of the whole group, level of care, a weekday/weekend indicator, and facility were included as fixed factors. When separated by level of care, adjustments were for weekday/weekend and facility. The number of days needed to obtain a reliability of at least 0.8 was estimated using the Spearman Brown prophecy formula:

*N* = *ICCt*/(1 − *ICCt*) × ((1 − *ICCs*)/*ICCs*), (1)

where *N* = number of days needed, *ICCt* = desired level of reliability, and *ICCs* = reliability for single days.

## 3. Results

Of 384 participants recruited, 257 (67%) agreed to wear the sensor. The remaining 127 residents either declined to wear the sensor or staff advised against it because of confusion and memory loss. Participant demographic information, cognitive profiles using the Montreal Cognitive Assessment (MoCA) [30] and mobility using the Timed Up and Go (TUG) assessment [31] are displayed in Table 1.

### 3.1. Number of Days Required

**Figure 2:**
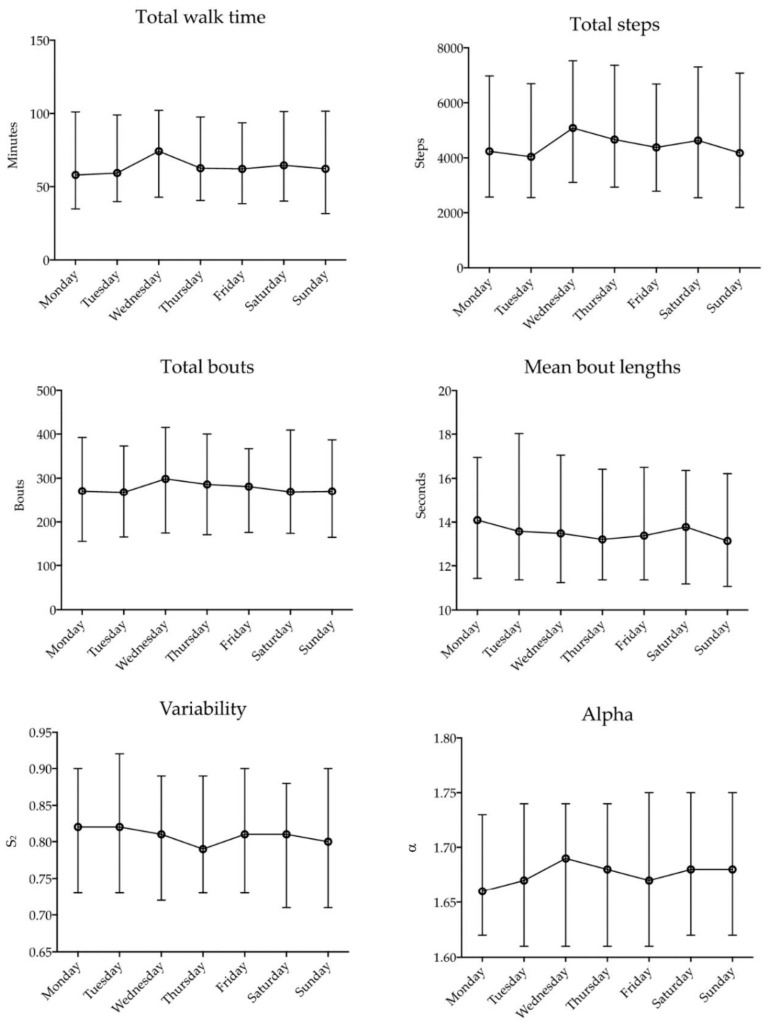
Median and interquartile range for total walk time, total steps, total bouts, mean bout lengths, variability and alpha for each day of the week.

At least 3 full days of data were recorded for 196 participants and were therefore included in the analysis of number of days required. When testing the impact of day of the week, post hoc analysis revealed no significant difference between any pairs of days (see Figure 2). There was a small but statistically significant difference in variability according to weekday/weekend, with slightly lower values recorded on average on weekend days (beta = −0.012, 95% confidence interval = −0.022 to −0.003, *p* = 0.013). Weekday/weekend was not a significant outcome for any other outcome measure.

For the whole group, the number of days required to achieve a reliability of at least 0.8 ranged from 2 to 5 days, depending upon the outcome. When stratified by level of care, the dementia level of care ranged from 1 to 3 days, the intermediate ranged from 2–7 days, and the high level of care ranged from 2–6 days (Table 2). Relative to volume-based outcomes, pattern and variability outcomes required a greater number of days to achieve an acceptable reliability.

Measurements from participants in the dementia unit demonstrated slightly lower reliability for total walk time, steps, and bouts compared to the intermediate and high care groups, indicating a greater within-participant variation in daily volume measurements for those in the dementia unit. Conversely, intermediate and high care groups demonstrated lower reliability for bout length, variability, and alpha, indicating greater within-participant variation in daily patterns of activity compared to those in dementia units.

### 3.2. Data Collection: Success Rate, Compliance, and Reason for Data Loss

**Figure 3:**
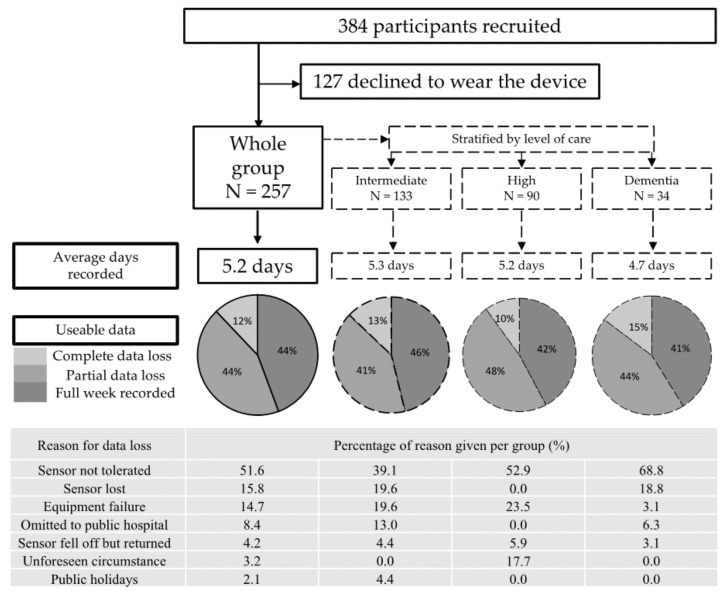
A flow diagram to describe the average number of days recorded, the percentage of full weeks recorded, partial data, or complete data loss. For the participants where a reason for data loss was provided, the reason for the data loss is displayed as a percentage for all explained data loss. The information is provided for all participants and also stratified by level of care.

Figure 3 shows the number of days of successful device wear, the reasons for data loss (where provided) for all participants, and individual care levels. Of the 257 participants, the target 7 days data (8 days wear time) was collected for 44% of participants, 44% recorded data for <7 days, and 12% did not return sufficient data (0 days recorded therefore complete data loss) for analysis.

Where information was provided, the majority of data loss was due to early removal of the wearable because of skin irritation or discomfort (51.6%), device loss, i.e., participants removing the wearable and, in some cases, losing it (15.8%), or equipment failure (battery discharge or software failure (14.7%)). Complete data loss was highest for the participants in the dementia level of care (15%). Dementia-level participants were also less able to tolerate the wearable for the full 7 days (68.8% of the reasons given were because of intolerance).

## 4. Discussion

This is the first study to determine how many days are required to obtain reliable physical activity walking outcomes for an ARC population with marked cognitive impairment, stratified by level of care. The average number days of activity recorded was 5.2 days for the whole group, and this was influenced by care level, with intermediate level of care wearing the sensor on average for 5.3 days, high level of care for 5.2 days, and the dementia level of care for 4.7 days. For the whole group, between 2 and 5 days were needed to achieve reliable measures of physical activity walking outcomes, and for people in all levels of care, the day of the week did not affect reliability for any of the outcomes calculated.

For all volume-related outcomes, i.e., total walk time, total step count, and total walking bouts, we found that two consecutive days of measurements were sufficient for a reliable representation on a group level using an ICC of 0.8. However, pattern and variability outcomes required between 3 and 5 days for the whole group. Comparing this to the average of 5.2 days of successfully collected data, it appears that the balance of obtaining sufficient data to reliably estimate walking outcomes was possible given the average wear time. When stratifying the group into different levels of care, the only exception to achieving this balance was for alpha, where the number of days required exceeded the average days recorded in the intermediate and high-level care groups. Previous examples have shown that volume, pattern, and variability do not always uniformly improve following intervention, but an improvement in a singular outcome can reflect a meaningful change [25]. For ARC residents, Barber et al. [2] speculated that shorter activity bouts should be promoted at the beginning of a physical activity intervention. If true, outcomes, that measure the proportion of short walking bouts, such as alpha, would be required to capture this intended intervention impact.

Comparisons between past investigations are problematic due to different sensor types, processing algorithms, sensor locations, outcomes, and participant heterogeneity. Despite these limitations, our findings agree with other recommended durations of data collection between 2 and 5 days [1,12].

Other researchers have recommended inclusion of at least one weekend day when measuring activity in adults [32,33]. However, we found no variance in activity between weekday and weekends in this ARC setting; meaning that inclusion of a weekend day is not necessary. Practically, this has advantages because it allows for more flexibility in administering the devices without the need to schedule specific days. Collectively, this may imply that, for an older population, if not bound to a typical weekday/weekend week structure, recording for 7 days may not be necessary.

The percentage of individuals who were willing to wear, or deemed by staff as able to wear, the device in our study was similar (67%) to that reported for an equivalent group of intermediate and high-level care residents, using a trunk-worn sensor (61%, *n* = 58) secured with a belt [11]. Successful wear was considerably higher than that reported for dementia residents using an ankle-secured device (29%, *n* = 127) [34]. By contrast, Resnick et al. [35] reported that 94% (*n* = 242) of residents in low level care were willing to wear a wrist-worn device (MotionWatch 8, CamNtech, Cambridge, UK). Key differences between our study and those noted above were the inclusion of older adults with significant dementia, the target duration of sensor wear (8 days wear versus 5 days [35] or 3 days [11]), the monitor location (low back, wrist, or ankle), the method of fixation (tape versus belt [11], wrist band [35], or ankle), and the variables measured (walking, spatiotemporal measures versus activity intensity [36]).

We chose to use a trunk-worn sensor because it allows more accurate step detection and walking parameters than a wrist-worn sensor, particularly at low gait speeds [36], as is the case in older adults. For our purposes, step detection rather than activity intensity (which is the output for the MotionWatch 8) was essential. Additionally, previous work using the AX3 worn in this manner have shown excellent compliance in older adults [21]. The benefit of the AX3 secured using tape is that it is waterproof, so does not need to be removed and replaced each day, which is problematic in ARC settings because of limited staff availability. However, the potential for skin irritation in this frail group of older adults is an issue and the main reason for early removal of the AX3 in 51.6% (*n* = 132) of cases in our study. In a few cases, dementia care residents removed the sensor themselves, possibly because it was bothering them or they did not know what it was. Similarly, Resnick et al. [35] reported that a small number of wrist-worn sensors were lost or removed by participants cutting or breaking the bands.

A strength of this study was the inclusion of a large number of participants with significant cognitive impairment and their stratification based on care needs. The results showed that the level of care impacted the number of days required in addition to the success rate of data collection. These results therefore support that future analysis should treat care levels separately for data analysis [2]. Interestingly, we found that the dementia level of care group, relative to the other levels of care, required less days for reliable measures of alpha and variability. Potentially, this could be due to a wandering trait [34], thus impacting the pattern and variability of walking and reducing between participant variance (i.e., the wandering behavior creating an increased amount of walking being common within the group). As such, due to the relationship between the chosen outcome measures and a trait that determines the level of care required, we recommend that, for outcomes quantifying walking outcomes, participants who require the dementia level of care should be treated as an independent group from other levels of care who do not exhibit wandering behavior.

Currently, there is no consensus for which is the best sensor, algorithm, and location for the measurement of gait/physical activity for this population [1]. For this population, many algorithms are yet to be validated [5], thus limiting the calculation of additional gait parameters, such as spatial gait information. Future research, similar to previous work achieved with Parkinson’s disease [19,20], is warranted to validate algorithms specific to the ARC population in an attempt to yield the potential value provided by discreet spatiotemporal gait characteristics. A limitation of this study was that a repeated week of analysis was not performed. This enables the ability to calculate how many days are required in addition to minimal detectable change on both a group and individual level [1]. Future research is warranted to examine the tolerance of different sensor locations without compromising the robustness of outcomes that reflect, for example, change over time or change in response to an intervention. Future work should also consider machine learning methods with the addition of complementary bio-signals (e.g., EMG) to improve walking detection and gait segmentation methods in this population [37,38].

## 5. Conclusions

This study showed that for a population of ARC participants of mixed levels of care, the average days recorded was 5.2 days. There was no impact of day of the week and, depending the outcome of interest, the required number of days ranged from 2 to 5 days, thus was within the average days recorded. The level of care did impact compliance to wear the sensor and the number of days required. This investigation therefore provides evidence capable of informing both future research and interventions aiming to quantify and improve physical activity walking outcomes, respectively.

## Figures and Tables

**Table 1:** Participant demographic information, cognition, and mobility scores.

Level of Care	Age (Years)	Weight (kg)	Height (m)	MoCA ^1^ (/30)	TUG ^2^ (s)
Whole group	84.54 ± 7.21	68.59 ± 17.66	1.62 ± 0.11	13.05 ± 7.63	30.13 ± 18.93
Intermediate	84.97 ± 7.55	70.4 ± 18.55	1.62 ± 0.11	14.29 ± 7.03	27.47 ± 17.26
High	85.04 ± 6.71	67.22 ± 15.1	1.62 ± 0.10	14.77 ± 7.29	35.53 ± 19.48
Dementia unit	81.53 ± 7.07	65.36 ± 11.79	1.63 ± 0.10	3.74 ± 5.04	26.01 ± 15.59

^1^ Montreal Cognitive Assessment (MoCA); ^2^ Timed Up and Go (TUG).

**Table 2:** The amount of days required for reliable gait outcomes indicative of walking volume, pattern, and variability for the whole population and separated by level of care.

Outcome		Level of Care	Number of Participants	Single Day ICC (95% CI)	Days Needed to Achieve ICC of 0.8
Volume	Total walk time	Whole group	196	0.71 (0.65–0.75)	2
		Dementia Unit	24	0.63 (0.46–0.77)	3
		High	68	0.70 (0.61–0.77)	2
		Intermediate	104	0.67 (0.59–0.74)	2
	Total steps	Whole group	196	0.70 (0.65–0.75)	2
		Dementia Unit	24	0.65 (0.49–0.79)	3
		High	68	0.69 (0.60–0.77)	2
		Intermediate	104	0.65 (0.57–0.72)	3
	Total bouts	Whole group	196	0.77 (0.73–0.81)	2
		Dementia Unit	24	0.67 (0.51–0.80)	2
		High	68	0.74 (0.66–0.81)	2
		Intermediate	104	0.75 (0.68–0.80)	2
Pattern	Mean bout length	Whole group	196	0.72 (0.68–0.77)	2
		Dementia Unit	24	0.76 (0.62–0.86)	2
		High	68	0.53 (0.43–0.64)	4
		Intermediate	104	0.56 (0.48–0.65)	4
	Alpha	Whole group	196	0.46 (0.40–0.53)	5
		Dementia Unit	24	0.75 (0.60–0.85)	2
		High	68	0.43 (0.32–0.54)	6
		Intermediate	104	0.37 (0.29–0.47)	7
Variability	Variability	Whole group	196	0.63 (0.57–0.68)	3
		Dementia Unit	24	0.80 (0.68–0.89)	1
		High	68	0.53 (0.42–0.63)	4
		Intermediate	104	0.49 (0.40–0.57)	5
